# Health promotion for the unemployed: the evaluation of the JOBS Program Germany from the trainers’ perspective

**DOI:** 10.1186/s13690-023-01203-2

**Published:** 2023-11-17

**Authors:** Heiko J. Jahn, Dennis Mayer, Alfons Hollederer

**Affiliations:** Section of Theory and Empirics of Health, The Faculty of Human Sciences, University of Kassel, Arnold- Bode-Str. 10, 34127 Kassel, Germany

**Keywords:** Unemployment, Mental health promotion, JOBS Program, Intervention, Randomized controlled trial, Germany

## Abstract

**Background:**

The JOBS Program Germany is a labor market integrative and health promotion intervention for unemployed people. This study examines JOBS Program Germany trainers’ view of (1) the theoretical concept of the JOBS Program, (2) its practical implementation in Germany on-site, (3) its acceptance by participants, and (4) the training effects. The study aimed at identifying potential for adaption allowing adjustments to improve the practical implementation and the training effectiveness.

**Methods:**

JOBS Program Germany trainers (two for each training) were interviewed via voluntary survey (computer-assisted web interviews) after each training.

**Results:**

Fourteen JOBS Program trainings have been conducted and all trainers responded resulting in 28 interviews. 78.5% and 85.7% of the respondents were rather or very satisfied with the theoretical contents and its practical implementation, respectively. Almost all trainers (96.4–100.0%) were satisfied with the on-site coordination, the cooperation with the organizer’s employees, the room equipment, the training room size, and the environmental conditions in the training rooms. In 89.3% of all responses the trainers rated the last training a success. However, the trainers also provided valuable suggestions for further improvement in Germany. This concerns the revision of the training manual, the adjustment of the trainer training and the preparation of participants.

**Conclusion:**

Besides the trainers’ positive view on the different dimensions of the training content and implementation, their suggestions can help ensure that many unemployed people in Germany could benefit from a continued regular implementation of the JOBS Program Germany in the future.

**Trial Registration:**

German Clinical Trials Register (DRKS), DRKS00022388. Registered on 20 July, 2020.

**Supplementary Information:**

The online version contains supplementary material available at 10.1186/s13690-023-01203-2.

**Table:** 

Text box 1. Contributions to the literature	
•Despite a substantial international body of literature on the “JOBS Program” intervention, this evaluation includes the assessment of the JOBS program trainers for the first time. This additional perspective enriches the state of research on labor market integrative health promotion and offers an additional perspective for the development of JOBS program, in Germany and internationally. •This paper reveals for the first time that also in the perspective of certified trainers, unemployed people benefit from JOBS Program. •These results indicate – in line with the available evidence – that JOBS Program should be constantly implemented into practice of health promotion among the unemployed.	

## Introduction

Many demands are placed on people who are faced with job loss or have already lost their job. This concerns, for example, dealing with fewer financial resources and often a loss of social relationships must be mastered [1–3]. Further, everyday life has to be redesigned and restructured [1, 4, 5]. In addition, family conflicts can arise, and new domestic roles usually have to be found [6]. Finally, unemployed people also often have to learn to cope with social stigmatization [7] and with experiences of failure during the application process [1, 8]. Such demands can lead to psychological distress and the empirical evidence for the association between unemployment and mental health impairments is well established [9–12]. Conversely, studies show [13, 14] and also the unemployed are aware of [1], that health problems represent barriers to (re)integration into the labor market – a vicious circle [8, 15, 16].

One intervention to improve the mental health of unemployed people is the so-called “JOBS Program”. This intervention (in the following called “JOBS training”) was developed in the 1980’s at Michigan Prevention Research Center of the Institute for Social Research, University of Michigan, USA [17]. It has been applied in different countries around the globe and was evaluated with positive effects in terms of (re-)integration into the labor market and/or mental health, for example, in the United States [18–21], in Finland [22, 23], Israel [24], Ireland [25], in the Netherlands [26], China [27], and South Africa [28].

In Germany from 2020 to the end of 2022, JOBS Program was offered within a German-wide pilot intervention in order to evaluate whether the same positive effects could be found in Germany and whether the JOBS training is also institutionally-wise transferable to the German conditions. The original JOBS Program was conceived as a preventive measure, primarily for the short-term unemployed, who have recently lost their jobs. However, in international comparison the unemployed population in Germany is characterised by a particularly high figure of long-term unemployed [29, 30]. This high stock of long-term unemployed is also in good economic times difficult to reduce, because it contains a high percentage of people with multiple barriers for (re)integration into the labor market, like older age, low qualifications or linguistic deficits [31].

The pilot introduction of the JOBS Program in Germany was imbedded into an inter-departmental cooperation framework called “Linking of Employment Promotion and Health Promotion in the Community Setting”. Hereby the Federal Centre for Health Education (BZgA) and the National Association of Statutory Health Insurance Funds (GKV-Spitzenverband) cooperated with the German Federal Employment Agency (BA). Within the framework of this pilot introduction in Germany, the JOBS training sessions lasted approximately 20-hours. The training was conducted within one or – in exceptions – max. two weeks in groups of 8 up to 15 participants and were led by two trainers [32]. This tandem of trainers should usually consists of two trainers: one of them usually works in the employment service or in adult education, typically without any experience of unemployment (”professional trainer”). The other trainer should be unemployed and would typically have no experience in job placement or adult education (“non-professional” or “peer trainer”) [32]. This team composition should, on the one hand, bring specialist knowledge about training/education and, on the other hand, the experience of unemployment into the training situation. Both have to undergo a specific workshop to be certified as JOBS Program trainer [32]. This workshop followed the described trainer training in the original JOBS Program Manual [17].

The JOBS training is mainly based on cognitive behavioral therapy methods with a focus on strengthening individual resources like self-esteem [33] and self-efficacy expectations [34]. The training uses active training methods (e.g., role plays) and aims also on increasing social support among the participants as well as between participants and trainers [17]. The trainings strive to create a sustainable motivation for job-search and to prepare the participants against setbacks during the job-search process (inoculation again setbacks) [17]. In sum, the training aims at improving the participant’s mental health and their chance for (re-)employment [17].

The University of Kassel conducted the outcome and process evaluation of the pilot introduction. A randomized controlled trial examined the effectiveness of the JOBS training concerning the influence on mental health and employment [35]. The detailed study protocol of this RCT was published [36]. Besides this, a formative evaluation, among those responsible for the initiation and organization of the local implementation of JOBS Program, within the framework of the overarching project for the interlinkage of health and employment promotion was conducted and published [37]. As another part of the formative evaluation, we interviewed the JOBS Program trainers and organizers via semi-standardized online survey, which is reported in this paper.

As a complementary element of the larger confirmatory evaluation of the JOBS Program Germany, the present survey aimed to get the perspective of the JOBS trainers on various aspects of the pilot implementation of the JOBS Program Germany. This concerns (1), the certified trainer workshop, (2) the revised German training manual, (3) the training methods and contents, (4) their feasibility and (5) in particular the training effects. In terms of a formative evaluation, we also wanted to find out (6) how the cooperation with the organizers on site worked and (7) whether the training conditions on site were appropriate. We also wanted to investigate (8) to what extent the unemployed participants accepted the JOBS trainings and the trainers and (9) whether the unemployed participants persevered through the trainings. In this way we wanted to evaluate the training and its practical implementation in Germany and identify the strengths and potential for improvement.

## Methods

### Data collection

After each training session between September 2021 and December 2022, we invited the two trainers by email to take part in an online survey using the online survey tool “LimeSurvey” (www.limesurvey.org). The email to the trainers contained a link which allowed access to the questionnaire of the survey. The survey was voluntary and the trainers had to give their written consent to participate before the survey.

We applied a semi-standard design collecting quantitative and qualitative data, through an online questionnaire. In addition to socio-demographic data, the questionnaire surveyed the respondents’ satisfaction or agreement with certain statements by use of Likert-type scales. Besides this, we asked the trainers to provide qualitative free text information regarding all possible aspects of the JOBS Program concept and its practical implementation, including administrative difficulties. In this way, we hoped to obtain additional qualitative information beyond the quantitative queries that could serve to further improve trainer education, training delivery, and administrative processes.

If a trainer had already led more than one training session (and was therefore already interviewed), questions concerning personal socio-demographic information and those that were more of a general nature (and did not explicitly refer to the last training session) were automatically omitted from the next survey. Therefore, we distinguished between (1) personal as well as general information collected only once (“general”) and (2) data on the trainers’ experiences concerning the last individual training session (“individual training”). The following information were collected.

#### Sample characteristics (general)

To examine whether personal characteristics might have influenced the evaluation of the JOBS Program training, we asked the trainers to provide socio-demographic information (age, sex, highest general education degree, and employment status).

#### Trainer workshop (general)

The workshop for the trainers is the crucial prerequisite to be able to conduct the JOBS training in its specific and predetermined way. The pilot workshop in Germany was led by Jukka Vuori [22, 23] according to the Finnish model of the JOBS Program. Therefore, in terms of formative evaluation, it is of particular interest whether the trainer felt well prepared by the trainer workshop and what they would suggest for improving the trainer workshop. The evaluation of the trainer workshop was operationalized using a self-developed five-point agreement Likert-type scale ranging from 1 (“completely disagree”) to 5 points (“completely agree”) with two items (see the single items and the Likert-type scale in Fig. 1 in the supplementary material).

#### JOBS Program training manual (general)

The most important tool for trainers is the JOBS Program training manual. It was originally developed by the Michigan Prevention Research Center mentioned above. On behalf of the BZgA, the training manual was initially translated verbatim and revised for use in Germany. The training manual was evaluated through a five-point agreement Likert-type scale ranging from 1 (“completely disagree”) to 5 points (“completely agree”) with seven items, which are illustrated in Figs. 2 and 3 in the supplementary material.

#### JOBS Program training (contents, implementation and effects) (general)

**Fig. 1:**
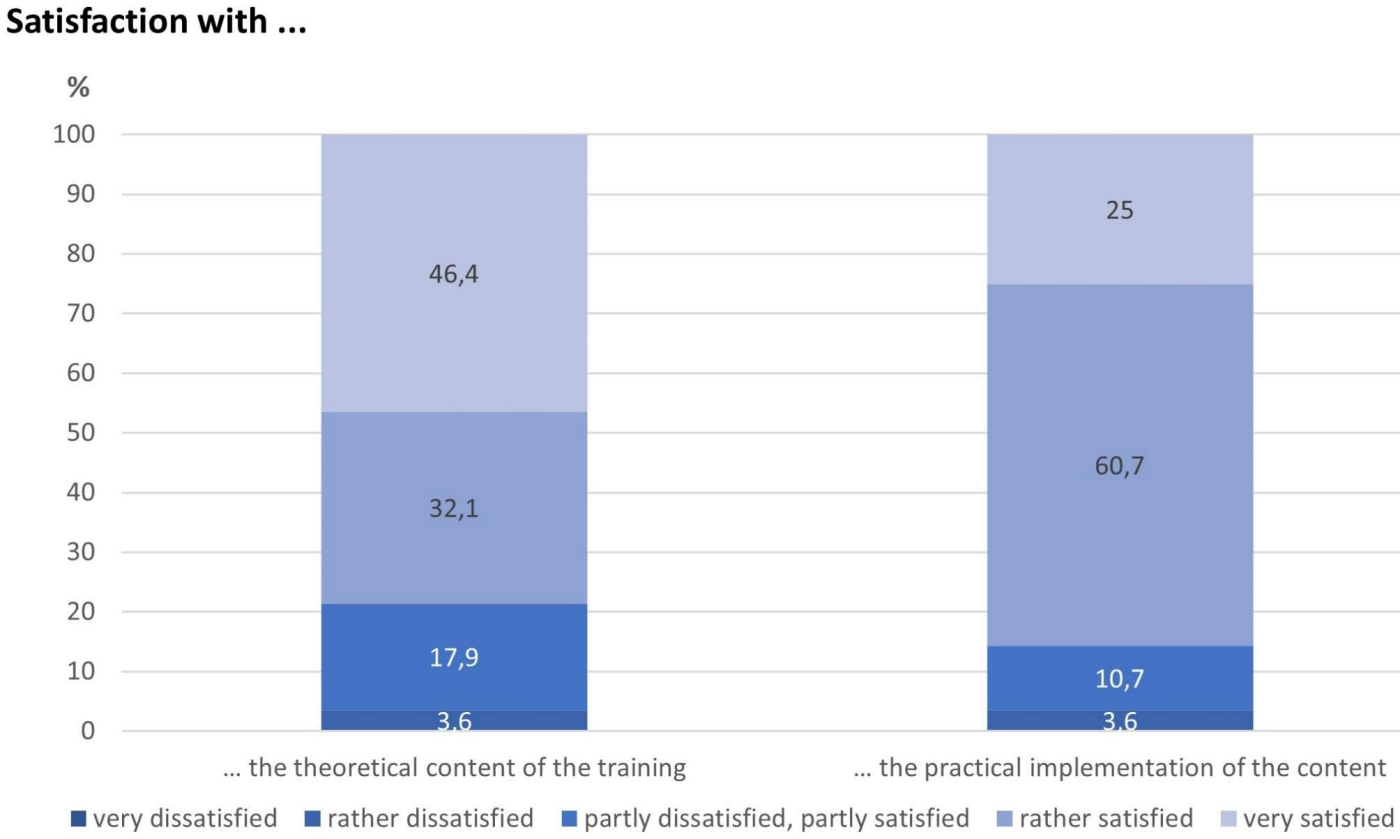
Satisfaction with the theoretical teaching content and its practical implementation (n = 26)

We asked the trainers for their general opinion on the theoretical content, of the training and the way the training was implemented and carried out in practice (e.g. timing, team composition). The items and the Likert-type scales to evaluate the training content and its practical implementation can be seen in Fig. 1 in the paper and in Figs. 4 and 5 in the supplementary material. In addition, the questionnaire contained items to measure the training effects (e.g. motivation for job-search, coping with setbacks) from the trainers’ perspective (see the single items and Likert-type scale in Fig. 7 in the supplementary material).

#### Training groups characteristics (individual training)

**Fig. 2:**
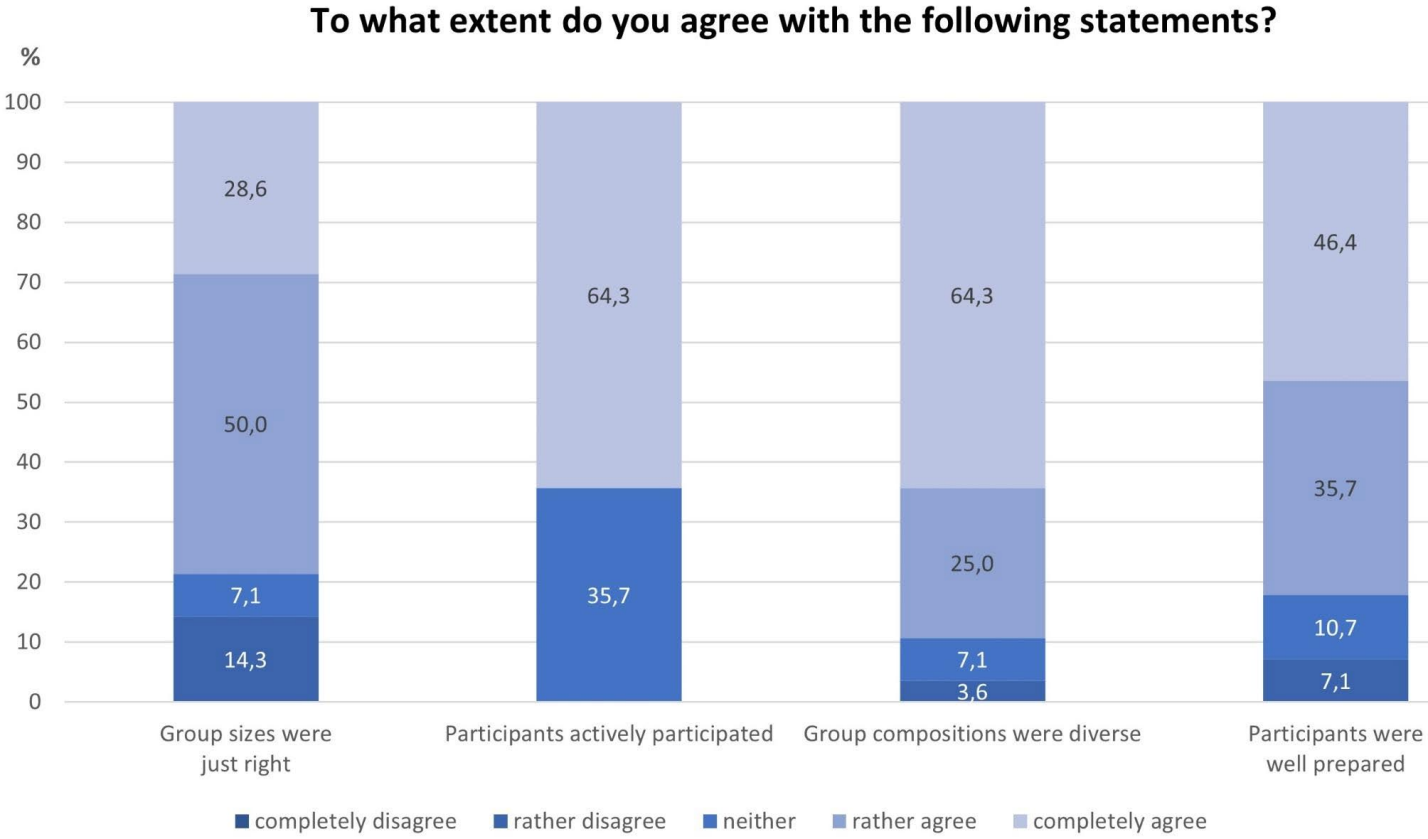
Trainer’s opinion about the characteristics and behaviors of the participants (n = 28)

The characteristics and behavior of the group can have an impact on the smooth delivery and success of the training. Therefore, we asked for some basic information about the group sizes, the diversity of the groups and whether the unemployed participated activeley in the training and were prepared for the training (see the single items and Likert-type scale in Fig. 2).

#### Training conditions and the on-site organization (individual training)

**Fig. 3:**
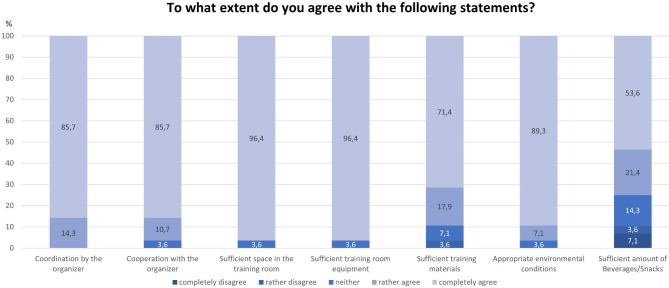
Satisfaction with the training conditions and the organization during on-site implementation of the JOBS training (n = 28)

We strived to examine whether the training organization and conditions were appropriate so that they would not impair the success of the training. This applies, to the coordination by the organizer, the cooperation with the local on-site organizers, the training room, the equipment in the training room, the environmental conditions, the training materials provided, the catering and the measures to protect against corona infections (see the single items and Likert-type scale in Fig. 3).

#### Comprehensibility of the training contents and its stimulation for cooperation (individual training)

**Fig. 4:**
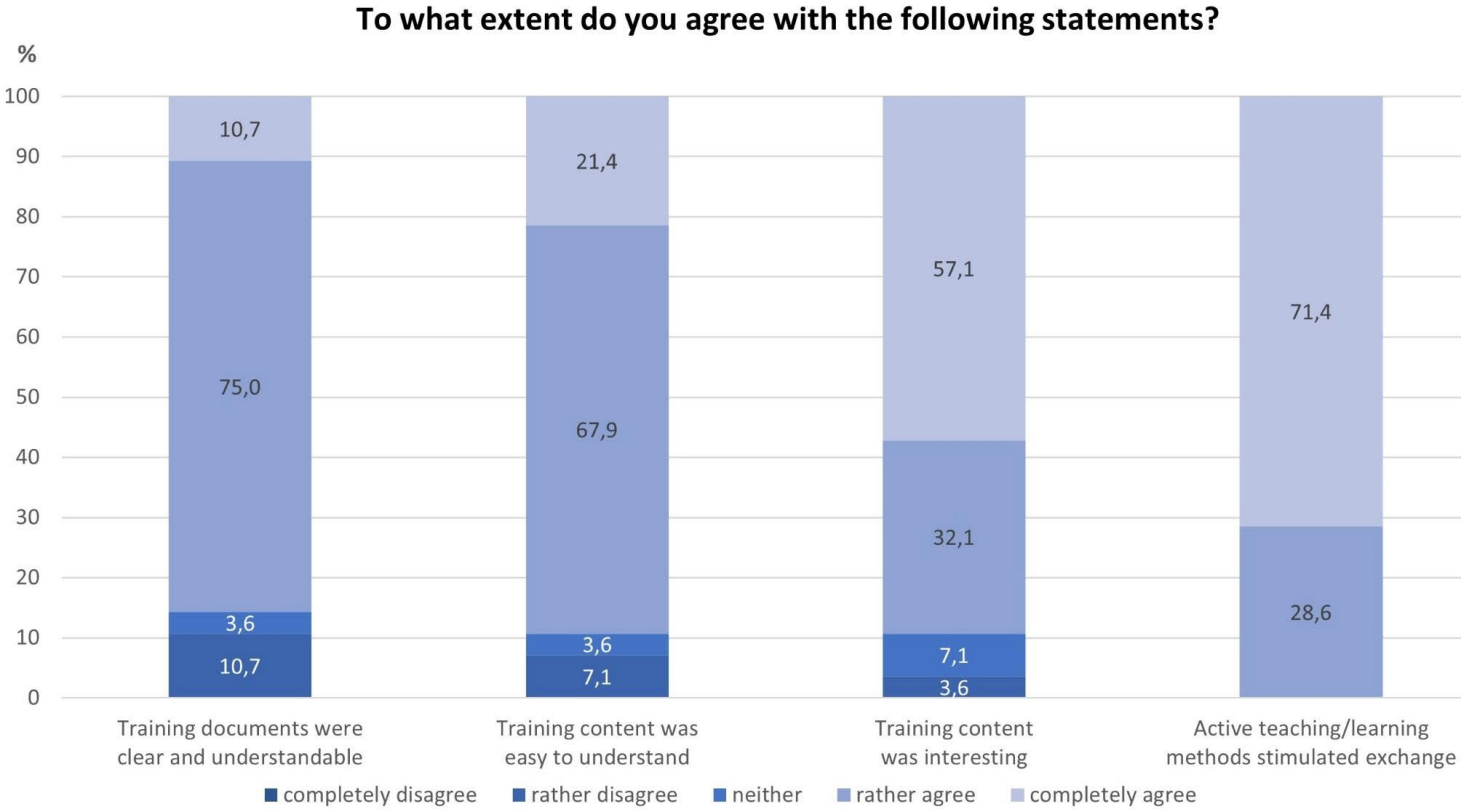
Trainer’s impression of the comprehensibility of training documents, content and active participation at each training (n = 28)

We also wanted to examine whether the participants easily understood the theoretical content and the training materials, which is an essential requirement for training success. In addition, we asked the trainers whether the active teaching/learning methods – an essential component of the JOBS training [17] – stimulated the desired exchange among the participants and between participants and trainers (see the single items and Likert-type scale in Fig. 4).

#### Referent power (individual training)

In order to achieve the greatest possible training success, it is important that the participants value the trainers personally and perceive them as reliable, professionally competent and approachable. In addition to creating a pleasant and motivating training atmosphere, the trainers strive – through their “referent power” [17]– to strengthen participants’ self-esteem and self-efficacy expectations. This can contribute to participants being more willing to view training content as valuable and motivated to implement it. In the JOBS Program training concept, “referent power” [17] is one of the core elements. Through competent appearance, self-revelation, the reduction of social distance and empathic support, trainers aim to gain a high level of appreciation, trust, and respect from participants [17].

**Fig. 5:**
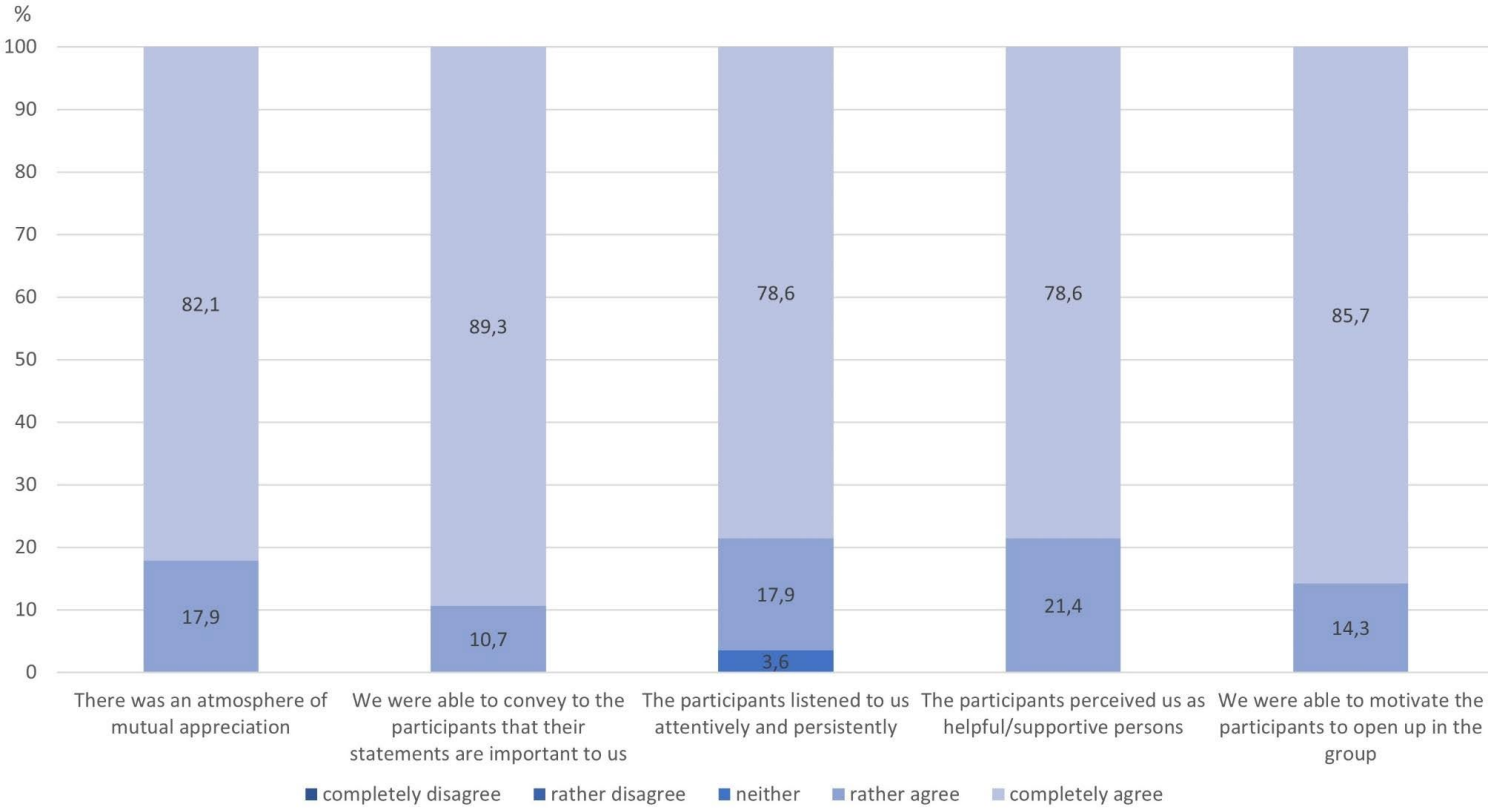
Items representing basic elements of referent power used to create a referent power sum-score (n = 28)

Referent power was operationalized using a self-developed five-point Likert-type scale ranging from 0 (“completely disagree”) to 4 points (“completely agree”) with five items. Since there is no standardized scale for referent power, we developed the items based on the strived referent power effects described in the original JOBS Program Training Manual [17] (see the single items in Fig. 5). We evaluated the single items and calculated a total score ranging from 0 (low) to 20 (high referent power).

#### Social support (individual training)

**Fig. 6:**
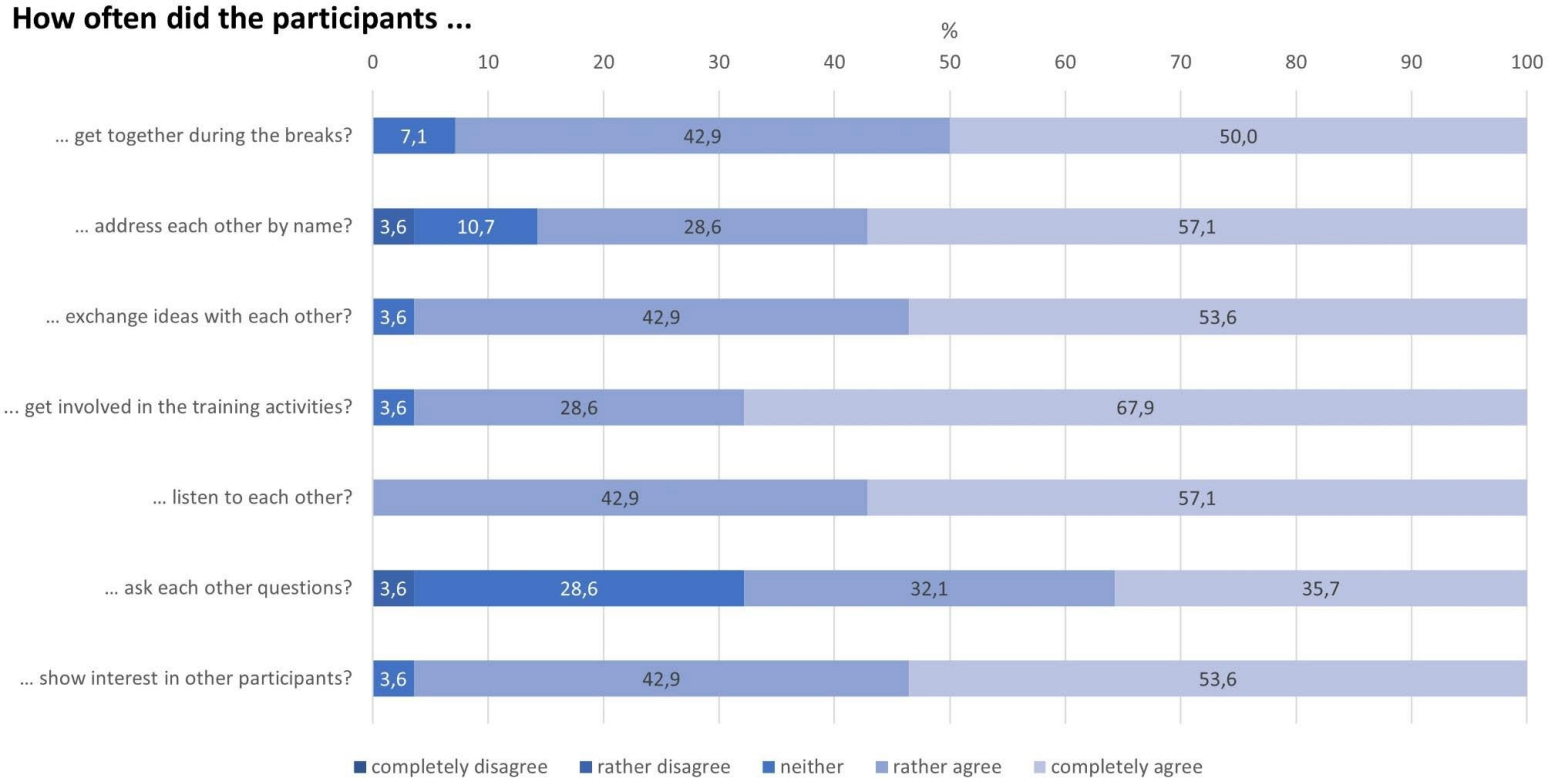
Items of dimensions of social support during the JOBS Program training (n = 28)

By using active teaching/learning methods and supporting feedback, the trainers strive to promote an appreciative and respectful interaction among the participants. The aim of this additional core element of the JOBS Program training concept is to create a positive group dynamic, which should also help the participants to support each other in the group exercises [17]. In the best case, this works beyond the actual training time in the sense of a sustainable supportive network. Social support was operationalized using a self-developed five-point Likert-type scale ranging from 0 (“never”) to 4 points (“always”) with seven items. Since there is no standardized scale for social support among training participants, we developed the items based on the goals for social support among participants formulated in the original JOBS Program training manual [17] (see the single items and Likert-type scale in Fig. 6). We evaluated the individual items and calculated a total score ranging from 0 (low) to 28 (high level of social support).

#### Self-efficacy (individual training)

Self-efficacy – another core element of the JOBS training – is an essential personal resource for coping with stress [34, 38]. It can be understood as “[…] the optimistic conviction […] of being able to cope with stressful demands based on one’s own ability and effort” [39]. Since unemployment can cause stress and thus impair mental health and the motivation to look for a job, both, [1] job-search specific and [2] general self-efficacy expectations and their improvement play an important role in the JOBS Program [17].

**Fig. 7:**
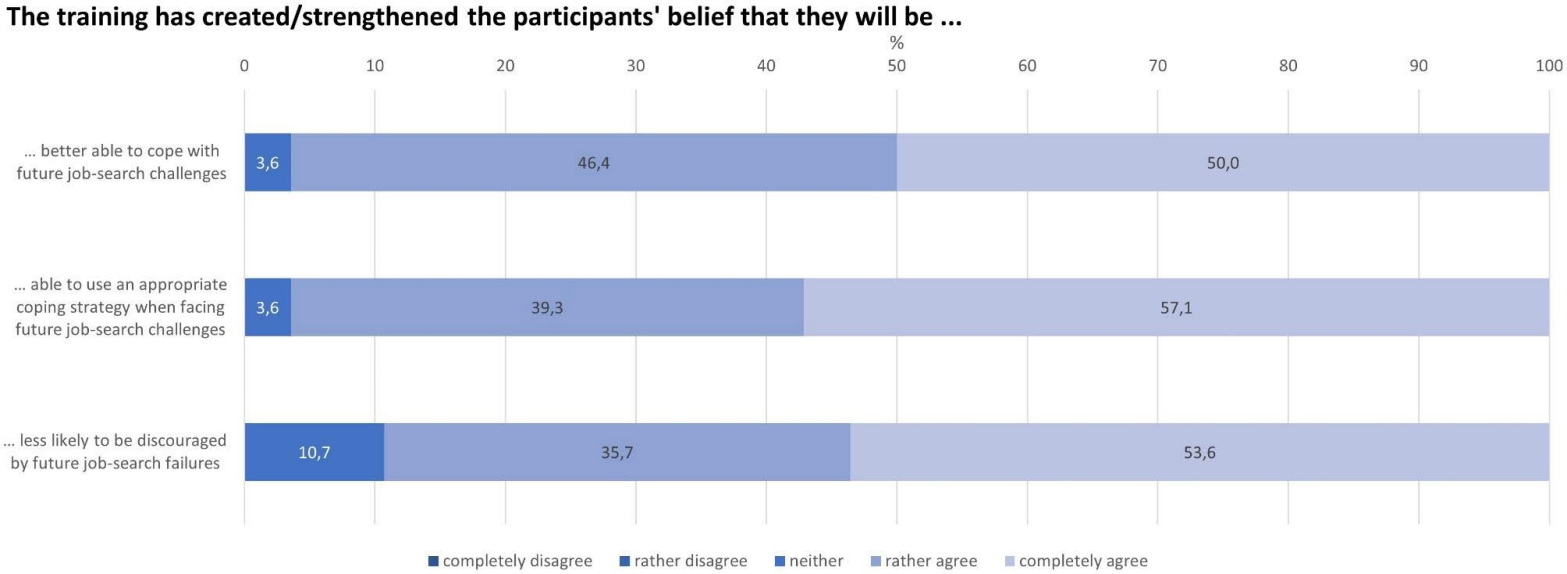
Participants’ job-search specific self-efficacy expectations with respect to actual coping abilities, appropriate coping strategies and inoculation against setbacks during job-search (n = 28; no trainer [rather or completely] disagreed)

Job-search specific self-efficacy were operationalized using a self-developed five-point Likert-type scale ranging from 0 (“completely disagree”) to 4 points (“completely agree”) with three items. We developed the items for Job-search specific self-efficacy expectations based on the formulated goals in the original JOBS Program Training Manual (see the single items and Likert-type scale in Fig. 7). We evaluated the individual items and calculated a total score ranging from 0 (low) to 12 (high level of job-search specific self-efficacy expectations).

**Fig. 8:**
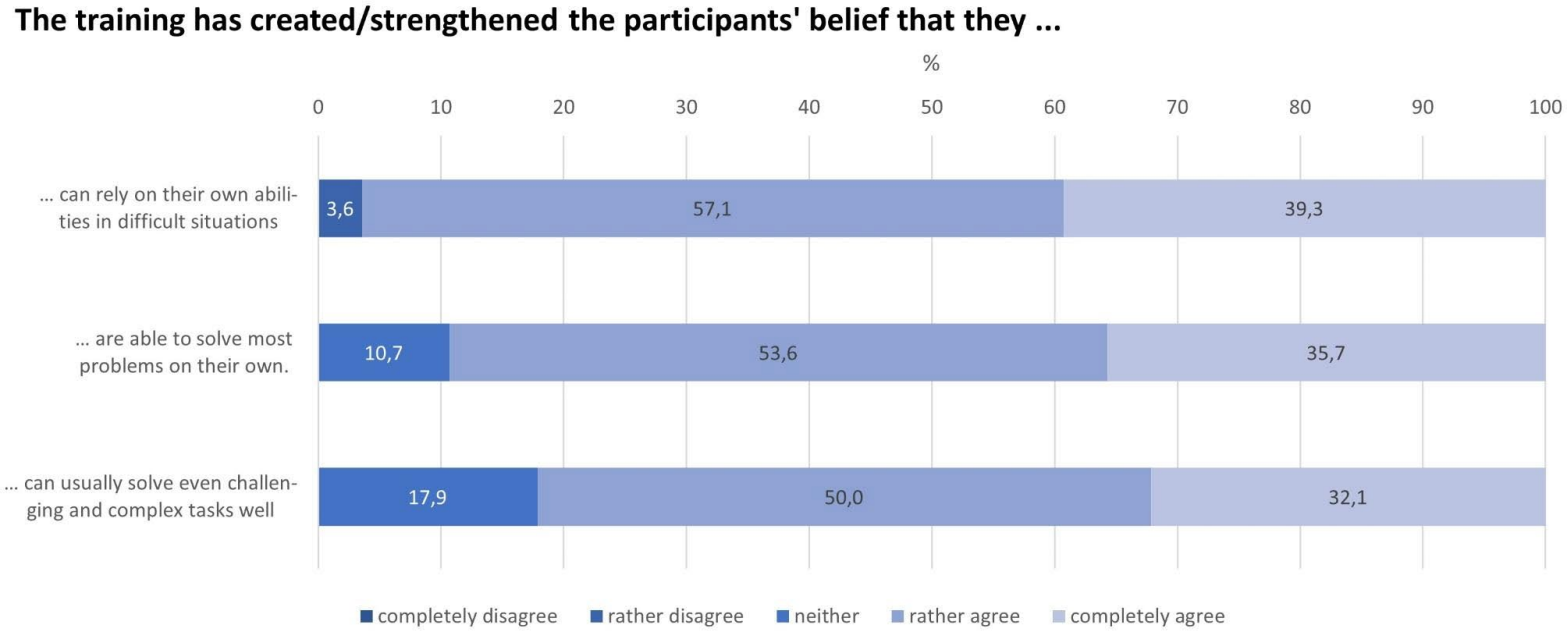
Participants’ general self-efficacy expectations with respect to actual coping abilities (n = 28; no trainer completely disagreed)

General self-efficacy expectations were also operationalized by using a five-point Likert-type scale ranging from 0 (“completely disagree”) to 4 points (“completely agree”) with three items. This scale was adapted from the General self-efficacy Short Scale (ASKU) [40] (see the single items and Likert-type scale in Fig. 8). We evaluated the individual items and calculated a total score ranging from 0 (low) to 12 (high level of general self-efficacy expectations).

#### Overall evaluation of the last training session (individual training)

In addition to the detailed aspects of the training, we wanted to know whether the trainers would assess their last training overall as successful or not. This was operationalized by the item “All in all, from your point of view, was the last JOBS training a success or a failure?” and a self-developed five-point Likert-type scale ranging from 0 (“a failure”) to 4 points (“a success”).

#### Evaluation of trainings from organizers’ view

As part of the formative evaluation of the JOBS Program Germany, we also interviewed the on-site training organizers by applying a short anonymous quantitative and qualitative LimeSurvey. The questionnaire differed from the trainer questionnaire and focused on the organizers’ experiences concerning the planning, organizing and practical implementation of the JOBS Program Germany as well as on their experiences in cooperation with stakeholders. We also wanted to know what role COVID-19 infection control measures played in this process and what suggestions the organizers have for improvement in future implementation of JOBS Program Germany.

### Data analysis

The data were analyzed using IBM SPSS Statistic Version 28.0. We carried out descriptive analyses and calculated sum-scores for the core training elements (referent power, social support, job-search specific and general self-efficacy). Based on the data measurement level and distribution characteristics, we applied Spearman correlations or non-parametric tests for independent samples to examine associations between these core elements and the above socio-demographic variables. A p-value of less than 0.05 was considered statistically significant.

## Results

In the following, the sample of the interviewed trainers is described first. In the results section, the results of the trainer surveys are reported, divided into results that relate (1) to the specific training courses carried out and (2) to results that relate in general to the JOBS Program and its practical implementation. Additional figures to the results that relate to specific training courses carried out are included in the supplement to the article.

### Sample characteristics (general)

Fourteen trainings were conducted between September 2021 and December 2022. All trainers participated in each online survey, resulting in 28 interviews. Six trainers conducted more than one training and were therefore interviewed multiple times. This results in 17 interviews that contain all responses and 11 interviews from the repeated surveys that only contain information on the most recently conducted trainings, but not on general questions e.g., on the training concept or on personal information (Table 1).

**Table 1:** Sample Characteristics

Person ID	Age (years)	Sex	Unemployed	Higher education entrance qualification	No. of interviews
1	60	Female	yes	No	1
2	34	Male	No	No	1
3	50	Female	Yes	Yes	1
4	36	Male	No	No	1
5	50	Female	Yes	No	1
6	36	Female	No	No	1
7	57	Male	No	Yes	2
8	41	Female	Yes	Yes	4
9	50	Male	No	Yes	1
10	58	Male	Yes	No	1
11	62	Female	Yes	Yes	1
12	55	Male	Yes	Yes	2
13	39	Male	Yes	Yes	2
14	41	Female	No	Yes	2
15	46	Male	No	Yes	1
16	54	Female	No	Yes	5
17	60	Male	yes	Yes	1
					Total = 28

On average, respondents (n = 17) were 48.8 years old and 52.9% were men. They were generally well educated, with almost two-thirds having a higher education entrance qualification (64.7%). In line with the training concept, slightly more than half of the respondents were unemployed (52.9%).

### Trainer workshop and JOBS Program training manual (general)

First, we interviewed the trainers (n = 17) about the trainer workshop. The majority of the trainers (n = 13) had after the workshop a clear idea of the theoretical content in terms of the underlying theoretical approaches as well as the teaching and learning methods and agreed (“rather” or “completely”) that they were aware of the objectives of the JOBS training. Also 14 trainers felt well prepared to put the specified training concept into practice (Fig. 1 in the supplementary material).

The free text answers show a more differentiated picture. We received eight responses on this topic. Most importantly, five times it was stated that more time should be spent on the trainer workshop, e.g.: “The workshop should run one day longer to be able to go into some topics in more depth”. One trainer suggested that at least the peer trainers should be better trained “[…] because for them the demands of running a course are already very high […]. I think a more intensive training of the peers (4–5 days) would be very helpful for this target group (strengthening self-confidence, building skills, better overview of the manual)”. Furthermore, it was suggested twice that the time constraints of the manual should not be strictly adhered to during the trainer workshop, and it was pointed out once that all exercises should be conducted and discussed during the trainer workshop at least once.

In addition, we asked the trainers about the content of the manual. All trainers (“rather” or “completely”) agreed that the content of the training manual contains everything needed to deliver the training. Besides this, 13 of the 17 interviewed trainers agreed (“rather” or “completely”) that the manual clearly explains how the training methods help the participants. However, six of the trainers rather disagreed that the manual’s content is easy to understand (Fig. 2 in the supplementary material).

In the free text responses, one trainer reported that the gender-sensitive language of the handouts had been difficult for participants to understand. Three responses referred to the timeliness of the manual: One trainer suggested that the manual should be “modernized” by including today’s online job-search options. Two further trainers argued along the same lines, writing e.g., that the “guideline for telephone applications is out of date”. One of those trainers went on to write that one should rather instruct how to use social media for job-search, how to create your own website with applicant profiles or applicant videos, e.g., with “story telling”. Other suggestions to improve the manual were to include a certificate of attendance as a handout that trainers could give to participants and that the frequently discussed aspect of “dealing with obstacles” should be worded differently so that it would be easier to find and collect solutions. Here, the problems would be often in the foreground.

We also asked the trainers about their opinion regarding the practical handling of the manual. Overall, the trainers seemed to be satisfied with the handling of the manual. Only two out of 17 trainers rather disagreed, that the manual is very helpful for carrying out the JOBS training (Fig. 3 in the supplementary material).

### JOBS Program training (contents, implementation and effects) (general)

#### Training contents

The trainers’ evaluation shows a high level of satisfaction with the training contents. For example only one trainer out of 17 rather disagreed that the training is useful for job-search and only one doubted that the training was suitable to improve the mental health of the participants (Fig. 4 in the supplementary material).

Overall, the trainers are relatively satisfied with the training content. However, some of them had different suggestions provided in the free text fields: One trainer suggested that more time should be allocated to identifying strengths and skills, as participants are “not used to […] being able to identify and name their resources as such […]”. The same trainer also suggested working out the everyday achievements of the unemployed, for example managing on a small budget, making sacrifices in order to make things possible for others, or dealing with crises. In addition, depending on the group composition, the “killer criterion” of single parenthood should be addressed, e.g., single parents’ organizational skills, their ability to work under pressure or taking on responsibility etc.

In addition, the topic of health was raised more frequently in the free text fields. For example, one trainer suggested paying more attention to the connection between health promotion, job search, stress management and social support. Another trainer advocated making participants more aware “[…] of their own health promotion, also with regard to future job prospects”. Three other responses stated that health was not visible enough as a topic, and one of those trainers went on to say that “[…] the connections between self-efficacy expectations and mental and physical health should be addressed more explicitly […]”. A sixth comment underscored this with the suggestion that the connection between unemployment and health should be better explained and that solutions should be developed based on the participants’ experiences.

#### Practical implementation

The training appears to have been designed to generate sustained interest among participants during the approximately five four-hour training days. For example, almost all trainers felt that both the methods (n = 17) and timing of the training (n = 16) were appropriate for maintaining participant interest. Furthermore, a large majority (n = 15) felt that the training was designed to allow the trainers to be responsive to the needs of the participants. In addition, it is again evident that the trainer composition targeted in the pilot launch of the JOBS Program was successful. Only one trainer could not clearly agree that the trainer tandems complemented each other well (Fig. 5 in the supplementary material).

Another aspect that is important for the practical implementation of the training is the time allocation for on-site implementation. Most of the trainers (n = 13) seem to be satisfied with the time allocated to conduct the training. On the other hand, four of the respondents disagreed or were undecided as to whether the time allocated was sufficient to adequately implement the training (Fig. 5 in the supplementary material).

This is consistent with free text comments: One trainer advocated for more time to conduct the training, especially if the educational differences within the groups are large and if there are language barriers. One trainer reported that participants wished “to extend the training to two weeks”. In addition to the total time allotted to complete the training, there were also comments about the timing of the single exercises. Two trainers reported that the time constraints for the exercises are sometimes calculated too tightly.

#### Trainer team composition

A large majority of the respondents (n = 16) preferred a team composition with two trainers, one of whom is a “professional trainer” (see above) together with a non-professional “peer trainer”. The second choice would be a team of two peer trainers (Fig. 6 in the supplementary material).

All the trainers were also asked to indicate whether they were able to work well with their tandem partners during the last training. Only one trainer (“rather”) disagreed that the cooperation was good. In all other surveys, the trainers (“rather” or “completely”) agreed that the cooperation between the trainers had gone well.

In the free text answers, it was indicated twice that the experience of having two trainers was beneficial to the trainers. One wrote that this way “[…] one of them could already see what task was coming next, while the other trainer presented the current task. This allowed the trainers to take a short mental break in between and then prepare for the next task”. One professional trainer reported a particularly good experience with a peer trainer, which underlines the importance of peer trainers in JOBS training: This peer trainer had a new job in prospect at the time of the training and thus also gained new motivation. The trainer wrote that through this experience of the peer trainer “[…] a very great transfer of motivation to the participants […]” could be observed.

On the other hand, the same professional trainer also reported that another peer trainer was demotivated by his own long-term unemployment during another training. During this training he doubted the solutions offered by the JOBS training and was therefore not always able to find his supporting role as a motivating trainer. The reporting professional trainer therefore suggested that it would be better to find peer trainers who are not affected by experiences of long-term unemployment. One peer trainer suggested that peer trainers should be involved in the planning and organization of the training as they know better the living conditions of the unemployed. It happened, for example, that training dates were set at the end of the month. According to the respondent, a peer trainer would have known that this could discourage unemployed people from attending the training because they would be more likely to spend the little money available at the end of the month on things other than tickets to the training site.

#### Trainers’ overall evaluation concerning content, implementation and effects

78.5% and 85.7% of the respondents were (“rather” or “very”) satisfied with the theoretical contents and its practical implementation, respectively (Fig. 1).

Having a closer look on the trainers’ assessment concerning the benefits for the participants offers a more detailed picture. For example, all trainers (“rather” or “completely”) agreed that the training empowers and motivates participants to lift themselves out of unemployment. 15 out of the 17 trainers also believed that the training would help the participants to find a job and 13 were the opinion that it helps them to cope better with setbacks during job-search (Fig. 7 in the supplementary material).

People without jobs are often unaware of the connection between unemployment and health. For this reason, health topics are also addressed in the JOBS training in order to sensitize the participants to this issue. However, four out of the 17 interviewed trainers did not (“rather” or “completely”) agree that the actual training concept conveys the connections between health and job-search.

### Training groups characteristics (individual training)

On average, the trainers reported that 6.3 participants appeared on the first day and 5.4 participants on the last day of the JOBS training, an average loss of 14.3%. The trainers found the group sizes to be good: In 78.6% of all responses (n = 28), they (“rather” or “completely”) agreed that the group size was “just right”. This is also confirmed by a qualitative free text response where one trainer wrote “Groups of up to nine people are ideal […]”.

In all 28 responses, the trainers (“rather” or “completely”) agreed that the participants got involved in the exercises and actively participated in the training (100.0%). In 89.3% of the responses the trainers (“rather” or “completely”) agreed that the participant groups were mixed in terms of various characteristics such as age, sex, migration background and length of unemployment and that the participants were well prepared for the JOBS training (e.g., through the information event, information from employees of the employment agency/job center, flyers, etc.) so that they could easily participate in the training (82.1%; Fig. 2).

### Training conditions and the on-site organization (individual training)

Overall, the trainers were very satisfied with the general conditions on-site. All respondents (“rather” or “completely”) agreed that they were satisfied with the on-site coordination (100.0%). 96.4% of the respondents (“rather” or “completely”) agreed that they were satisfied with the cooperation with the organizer’s employees, with the room equipment (such as tables and chairs), with the training room size as well as with the environmental conditions in the training room (e.g., temperature, ventilation, lighting, noise). A large majority were also satisfied with the amount of training materials (e.g., flip charts, pens, tape, etc.; 89.3%) as well as with the beverages and snacks (e.g., candy, cookies, etc.) (75.0%) provided (Fig. 3).

Despite the good overall results, there were also suggestions for improvement in the free texts: A trainer wrote that it would have been “[…] desirable if they had got materials such as binders, writing pads and pens available to the participants. This would be an effective sign of appreciation. So we, as trainers, bought these materials from our private fortune”. Two trainers suggested that the organizer should provide more drinks and biscuits and it was once stated that more material such as flip charts would be good.

#### Corona infection protection measures

In 23 of the 28 responses, it was indicated that infection control measures were mandatory during training (In 11 of the 14 trainings [78.6%]). In nine of these 23 responses, the trainers (“rather” or “completely”) agreed that the infection control measures affected the implementation of the trainings (39.1%).

In the free text answers in this regard, it was stated nine times that the masks had impeded communication. This related to speaking and facial expressions as well as understanding spoken content. Furthermore, it was reported twice each that the training room became too cold due to the ventilation and that certain exercises could not be carried out due to the infection control measures. Additionally, the exercises needed more time to maintain personal distance, but it was not always easy for the trainers to implement all the measures. One wrote: “[We] did ask the participants to pay attention to spacing, but it was not always respected”.

### Comprehensibility of the training contents and its stimulation for cooperation (individual training)

In line with the results above, the trainers also (“rather” or “completely”) agreed that the active teaching/learning methods stimulated the exchange among the participants (100.0%). Overall, the trainers assessed that the training documents and contents were understandable and the latter also interesting for the participants. However, it seems the training content was better understandable than the training documents (i.e. primarily the worksheets that are part of the training manual) (Fig. 4).

### JOBS program core elements (individual training)

#### Referent power

The trainers felt, they were able to generate a high level of referent power during the trainings. The mean sum-score for all five items was 19.11 (median = 20) out of a maximum of 20 possible points. In four out of five Items considered here to represent the referent power, in all 28 responses the trainers (“rather” or “completely”) agreed that the addressed aspect was fulfilled. Only concerning the statement “The participants listened to us attentively and persistently”, one trainer was undecided once (Fig. 5). There were no associations between socio-demographic variables and the referent power sum-score.

#### Social support

The results of the single items show that, from the trainers’ point of view, it was possible to create a situation of social support. The trainers had the impression that the participants not only worked well together in the group exercises and role-plays, but also showed personal interest in each other and exchanged ideas, even beyond the training sessions during the breaks (Fig. 6).

The social support sum-score with a mean of 24 points (median = 24) out of a maximum of 28 points achievable shows that the trainers rated the level of social support as high. There were no associations between socio-demographic variables and the social support sum-score.

#### Job-search specific and general self-efficacy

Most of the trainers reported that the training resulted in a high level of job-search specific self-efficacy expectations among the participants (Fig. 7). The total score for those three items was 10.4 (median = 11) out of a maximum of 12 possible points. There were no associations between socio-demographic variables and the job-search specific self-efficacy expectations sum-score.

An additional qualitative statement in the survey that directly related to participants’ job-search specific self-efficacy expectations and self-confidence (“Through the training, participants gained greater self-confidence/higher self-efficacy expectations for job search”) was largely confirmed: in 27 out of 28 responses (96.4%), the trainers (“rather” or “completely”) agreed that the participants were able to increase their job-search specific self-efficacy as well as their self-confidence through the training.

A similar positive picture can be seen when looking at Fig. 8. The majority believed that the training contributed also to an increased level of general self-efficacy. For instance, in only one out of 28 responses, a trainer rather disagreed that the training has created or strengthened the participants’ belief that they can rely on their own abilities in difficult situations.

The general self-efficacy expectations sum-score with an average of 9.7 (median = 9.5) out of a maximum of 12 points achievable indicate that the trainers believe that the training had a positive influence on the level of general self-efficacy expectations as high among the participants. There were no associations with the socio-demographic variables age, sex, and highest level of education. However, despite the small sample size, we found a statistically significant association between the trainers’ assessment of the level of participants’ general self-efficacy expectations and the trainers’ employment status. The mean value estimated by the peer trainers was higher than the value estimated by the professional trainers (9.9 vs. 8.5 points; p = 0.027).

### Overall evaluation of the last training session (individual training)

**Fig. 9:**
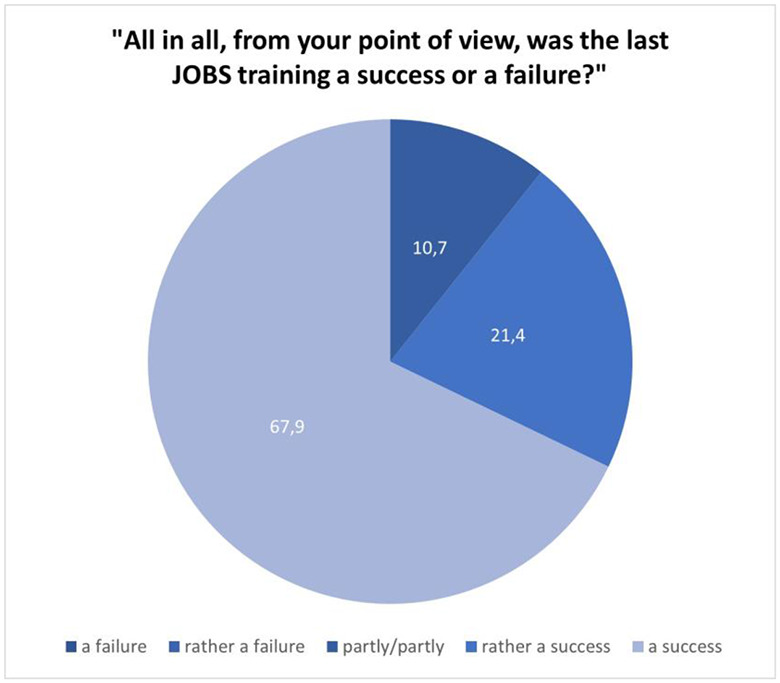
Overall success from the trainers‘ point of view (in %; n = 28; no trainer rated the last training as [rather] a failure)

The average overall rating was 3.57 (median = 4). In only three (10.7%) of 28 responses, trainers were unsure whether the last training session was a success or not. All other responses (89.3%) clearly reflect a very positive résumé (Fig. 9).

### Organizer evaluation

We also interviewed the on-site training organizers. Participation was voluntarily and anonymous. Four Job centers and one educational institution rated the practical implementation of the JOBS program as a success. Two organizers reported difficulties due to the infection control measures during the COVID-19 pandemic. There was a lack of participants because of the fear of infection, ban on participation for non-vaccinated persons, distance rules that hampered the training conduction etc. One organizer reported that short-term peer trainer cancellations hampered the participant recruitment.

Another point of criticism concerns the cooperation with the BZgA: One organizer did not feel sufficiently supported in the implementation of the training and criticized the high administrative effort of the cooperation as well as “[…] the difficult accessibility by telephone, the constantly changing contact persons and the poor flow of information”. Two other organizers also complained about the high bureaucratic effort and the poor flow of information.” One organizer indicated that better implementation of the training would require more lead time. In addition, this organizer suggested producing a video for the JOBS program Germany, which could be helpful in recruiting participants.

## Discussion

To the best of our knowledge, no evaluation of the JOBS Program training has ever used such a broad evaluation concept. This study is a part of it and investigated how the JOBS Program trainers evaluate the JOBS Program concept and its methods in terms of its impact on the participants. In addition, the trainers evaluated their trainer workshop, the training manual and the practical implementation of the training as well as the cooperation with the local organizers on-site.

### Trainer workshop

The evaluation of the trainer workshop shows a heterogeneous picture. The vast majority agreed that the trainer workshop had given them a clear idea of the theoretical approaches underlying the training, the teaching and learning methods, and the training objectives. In addition, the majority of trainers apparently also felt that the training had prepared them well to put this theoretical content into practice. In contrast, three of the trainers rather disagreed that they had a clear idea of the theoretical content and the goals of the training or that they felt well prepared.

Regarding the improvement of the trainer workshop the qualitative data provide deeper information: The trainers suggested several times that the trainer workshop time should be extended. Specifically, it was suggested that the workshop of the trainers should at least be extended to such an extent that all the exercises could be tried out by the trainee trainers to be well prepared to competently perform the exercises. Both trainers should have the opportunity during the trainer workshop to internalize all the exercises, for example, by performing them in the role of trainer and participant and reflecting on them afterwards in the trainer workshop group. We assume that the trainer workshop would have been rated better by the trainees under different circumstances. This is because the original trainer training concept called for more in-person instruction and follow-up sessions. However, due to the COVID-19 pandemic and the associated infection control measures, the already proven training concept could no longer be implemented, and more blended learning sessions had to be used.

However, in this context it should also be noted that the type of knowledge transfer during JOBS training plays a key role. Because, through their appearance and personal interaction with the participants, the trainers have a particular influence on the referent power and the social support. Therefore, the success of the training stands and falls with the workshop of the trainee trainers, both in terms of their personal communication with the participants and their competent implementation of the exercises. Consequently, the pre-pandemic trainer workshop concept should be implemented again in the future, even if it is more time-consuming and costly.

### JOBS Program training manual

Since the training manual is considered the heart of the training, to which trainers should adhere to in detail (sometimes to the point of phrasing positive feedback and appreciation for the participants, etc.), it is vital that the manual is easy to use, and the content is easy to understand. At first glance, the quantitative results do not seem too bad in this regard. However, six trainers rather disagreed that the manual’s content is easy to understand. Furthermore, the qualitative responses indicate that the manual needs to be further adapted to reflect current (e.g., online) job search and application methods. Given the importance of the manual, it seems necessary to further revise the manual before the JOBS Program training can be integrated into regular labor market integrative health promotion in Germany.

### JOBS Program training (contents, implementation and effects) (general)

#### Training contents

On the whole, the findings suggest that the trainers are quite satisfied with the training contents, their practical implementation, and the training effects. Nevertheless, the qualitative information in particular provides valuable indications for future improvements. In terms of content, these concerns, for example, the desire to devote more time to promoting self-efficacy expectations and teaching health aspects. The latter is also in line with the results of the trainers’ overall JOBS training evaluation (see discussion under “Trainers’ overall evaluation concerning content, implementation and effects”). These wishes of the trainers indicate that the concept of the JOBS Program is well received by them. This is because both aspects, self-efficacy and health, are of particular importance in the theoretical approach.

#### Practical implementation

Almost all dimensions of practical implementation addressed in this survey show that the trainers were very satisfied. This concerns the timing of the exercise, the cooperation between the two trainers, the methods, and the target group orientation.

However, one aspect that should be looked at more closely in the future is the training time, which was too short for some trainers to be able to convey the content and exercises satisfactorily in this group. Almost every fifth trainer (“rather”) disagreed that the training plan provides sufficient time for teaching the content. Perhaps the composition of the training participants also had an effect here: Since the majority of the training participants were middle-aged (mean = 44.7 years) and long-term unemployed (mean = 6.4 years) [35], this may have had an impact on the learning speed. There is reason to believe that the present training concept, with a duration of about 20 h, is more suitable for younger people or short-term unemployed participants. In most evaluation studies of JOBS Program, participants were younger and unemployed for less time. It might therefore make sense to create target group-specific adjustments. For example, the duration of the training could be varied in relation to the average age of the participant groups. Altogether, these results are promising and are in line with the overall evaluation of the individual trainings (Fig. 1). This suggests that the practical implementation will also work in future, but it must be acknowledged that the trainers also require sufficient time to convey the contents taught.

#### Trainer team composition

The results clearly show that the trainers prefer the combination of a peer trainer and a professional trainer by far. This is in line with a previous evaluation of the JOBS Program in Ireland [25]. Remarkable is, that both the peer trainers and the professional trainers attribute particular relevance to the peer trainers. This is to the point that, on average, trainers found it preferable for the training to be conducted by only one peer trainer instead of two professional trainers. The peer trainers are important, among other things, because they are familiar with the living conditions of the unemployed participants through their own unemployment experiences and it is therefore plausible that the participants feel a smaller social distance to the peer trainers than to the professional trainers. As participants may see the peer trainer more as ‘one of them’, they may conclude that the peer trainer can more easily empathize with the problems they face in their job search and daily life. This applies in particular to the topics that the JOBS training takes up, such as the often low self-efficacy expectations and the low self-confidence of the unemployed as well as the frustration that an unsuccessful job-search can bring with it. This social closeness and empathy between participants and peer trainers enables participants to feel understood – perhaps more than by professional trainers. This could help them to open up in the group, to accept the training content more easily and to actively participate in the training exercises.

#### Trainers’ overall evaluation concerning content, implementation and effects

As mentioned above, in general, the trainers were quite satisfied with the theoretical content of the JOBS Program (underlying theoretical approaches and teaching/learning methods) and with the practical implementation of this theoretical content (Fig. 1).

Additionally, the five further items evaluating most important benefits for the training participants (referring to re-employment and health aspects) show promising results as it is crucial that the participants consider themselves as capable of applying their newly acquired job-search skills and that they do not lose their job-search motivation, even if there are setbacks during job search.

However, it is striking that four trainers did not (“rather” or “completely”) agree with the statement “The training conveys the connections between health and job-search to the participants”, which is worst rated item in this context. This was also addressed by the qualitative information concerning the trainers’ opinion about the JOBS Program contents in general under Training contents. Several trainers highlighted the importance of the connection between unemployment and health, of health promotion and of other health-related aspects and some stated that more time should be allocated to address these topics during the training session. The repeated suggestions at various points in the survey underline the importance of these health-related topics and suggest that these aspects should be given greater attention in the further development of the JOBS Program in Germany and on international level.

### Training groups characteristics

The training groups were relatively small and the majority of the trainers were satisfied with this. However, more than one fifth did not agree that the group size was “just right”. We do not have specific responses to that, but it may also be that some trainers found the groups too small. There were seven trainings with fewer than six participants and four trainings even with fewer than five participants, which can make group work or role-play exercises difficult.

In the free text responses, however, it was said that the small groups were helpful and that it was good to be able to divide the work between two trainers in order to be able to adequately take care of the participants. Since most of the trainers had only conducted one or a few trainings, it would certainly be possible to increase the group sizes slightly as they gained experience with further trainings. However, the maximum group size of 15 persons, as described in the training manual [17, 32], should not be exceeded so that intensive support of the participants is possible.

Another possibility to improve the training delivery may be the preparation of the participants: Almost every fifth trainer felt that the participants were not well prepared for the training. This is not a very bad result, but it shows that there is still room for improvement. In this context, it is important to mention that the trainings were organized and conducted during the COVID-19 pandemic and this caused tremendous difficulties in recruiting and informing the participants. After the release of the infection control measures, it is very likely that, for example, employment agencies can better inform potential participants about the training. A “marketing” video about the JOBS Program, as previously suggested, could additionally help recruit participants.

An important criterion for the successful implementation of learning processes is the sustained interest of the learners. This was apparently achieved through the training concept. The participants have obviously been actively involved, and even throughout the entire course of the training sessions. When evaluating the training concept and the training implementation, almost all trainers (“rather” or “completely”) agreed that the training methods and the training timing (i.e. the temporal structure or sequence of the training) was suitable for maintaining the interest of the participants.

### Training conditions and the on-site organization

Cooperation with the local organizers of the training is also important for the smooth implementation of the training on-site. The organizers could be very different institutions, such as job centers, church-related social welfare organizations that offer educational services as well as state or commercial educational institutions. Fortunately, the trainers were on the whole satisfied with the organization of the trainings on-site as well as with the cooperation with the organizers’ staff. This is also important for this study. It shows not only that the JOBS trainings can be implemented well in practice, but also that the cooperation and organization probably had no negative influences on the training implementation and thus on its intended effects.

The trainers see some potential for improvement in the provision of catering and work materials. It can be concluded from both the quantitative and qualitative responses that some trainers would appreciate if the socio-economically less advantaged participants were provided with sufficient working utensils, such as pens, pads, etc., as well as with drinks and snacks. This went so far that two trainers privately financed such items.

Another difficulty was that the training was apparently affected by the SARS-CoV-2 infection control measures. As described, the distance between the participants and the frequent airing of the training rooms sometimes affected the exercises. However, this is not a problem specific to JOBS training. Rather, these complications have already been eliminated because the infection control measures have been removed.

### Comprehensibility of the training contents and its stimulation for cooperation

The findings indicate that the comprehensibility of the training contents, which is crucial for the training success, seems to be fulfilled and it should not be a high hurdle to revise the worksheets that were less comprehensible for the unemployed training participants. Based on our data, we cannot say for sure to what extent the worksheets were difficult to understand. Perhaps creating the worksheets in different languages for participants with some kind of migration history and in simple language versions would be sufficient to meet the needs of this target group.

### JOBS Program core elements (individual training)

Some of the most important elements of the JOBS training are the referent power, the social support among the participants (particularly during the training) and the participants’ job-search specific and general self-efficacy expectations [17, 32]. A high level of those elements should ultimately lead to better mental health, to higher and sustainable job-search motivation as well as to (re-)integration into the labor market [17, 32].

As the results of the mean sum-scores suggest, the trainers rate the achieved referent power and social support as high with 95.6% and 85.9% of the max. achievable points. The same applies to the positive effects on the level of job search specific and general self-efficacy expectations with 86.9% and 80.9%, respectively, of the max. achievable points. Having a closer look on the six items used to collect data on (job-search specific and general) self-efficacy (Figs. 7 and 8), The worst item-result was that 82.1% of the trainers were the opinion that the training has created/strengthened the participants’ belief that they are usually able to solve even difficult and complicated tasks. All other five respective items showed even better results.

Considering these positive results, the trainers are therefore obviously convinced that the training concept has been successful and that its implementation has worked well. This was also clearly reflected in the trainers’ satisfaction with the theoretical teaching content and practical implementation in general (Fig. 1) and in the overall positive evaluation of the last training session (Fig. 9).

The trainers’ positive assessment is confirmed by the results from our survey among their unemployed training participants that showed partly statistically significant association between the JOBS training and the participants’ self-rated job-search specific self-efficacy [35]. Further, we can confirm that previous JOBS Program evaluation studies also identified positive training effects on job-search specific self-efficacy [19, 26, 28, 41–43].

When looking at individual items, one can conclude that the trainers believe that the essential endpoints are achieved by the training. This applies to the high level of agreement with the statements from the summary evaluation that the training (1) helps participants to help themselves (increasing job-search specific self-efficacy), (2) motivates them to look for a job, (3) helps them to cope better with job search failures and (4) helps them to find a job. These statements are supported by the trainers’ assessment of the training content, (1) that the training can improve mental health and (2) provides appropriate strategies for dealing with job search setbacks.

For the core elements that we examined in more detail (referent power, social support, job-search specific and general self-efficacy), we did not find strong associations between their sum-scores and the socio-demographic characteristics of the trainers. On the one hand, this could be because the sample is small. On the other hand, the sum-score differences between the subcategories of the categorical variables (e.g., sex, education, employment status) were mostly moderate. A single statistically significant result emerged when comparing unemployed and employed trainers with respect to their assessment of the effect of the training on participants’ general self-efficacy expectations. The peer trainers’ responses led to a higher sum-score as compared to the professional trainers’ responses.

### Strengths and limitations

To our knowledge, this is the most comprehensive survey of JOBS Program trainers, who play a critical role in the success of the training. This survey was not only about practical implementation, but also about training effects. However, the latter must be considered against the methodological background that we did not measure the training effect directly. Rather, we were interested in finding out to what extent the trainers were satisfied with the implementation of the training and what effects they felt the training had achieved. This is a rather indirect approach and one cannot exclude that the personal views of the trainers differ from objective effect measurements. In addition, it must be taken into account that trainers partly evaluate their own work, which – consciously or unconsciously – may lead to biased responses. However, since the JOBS training is intensive and usually lasts five days at a time, we are confident that the trainers will get a good impression of the participants’ progress. This enables them to assess the training effects and thus provide important information for this study and for future decision-making.

Due to the cross-sectional character of the study design, the trainers cannot assess whether the positive effects are sustainable, e.g., regarding job-search specific self-efficacy expectations or inoculation against setbacks. However, it should be noted that this was not aim of this trainer survey but was examined in a randomized controlled trial among unemployed JOBS Program Germany participants reported elsewhere [35].

Another strength of this study is that, for the first time, we tried to evaluate the trainers’ perspective on the core elements of the JOBS Program, such as the referent power or the job-search-specific self-efficacy. The results are mostly positive and provide details that will be helpful to future trainers. On the other hand, there were no validated and free available instruments for those core elements. So, we were forced to develop the applied scales ourselves. Other limitations are the small sample size and the lack of a comparison group. The small sample is due to the almost impossible participant recruitment and implementation difficulties during the COVID-19 pandemic and the related infection control measures. A comparison group was not provided because the training effect was examined as part of the above-mentioned randomized controlled trial.

## Conclusions

To promote the health and reintegration of the unemployed into the labor market, an approach aimed at improving personal resources is needed. The JOBS Program Germany aims to strengthen such resources, including self-efficacy, inoculation against setbacks, and the promotion of sustainable motivation to find a job. Despite all the limitations due to the COVID-19 pandemic and its consequences for trainer training, participant recruitment and practical implementation in the field, this trainer survey shows a positive evaluation.

Overall, the trainers were satisfied with the theoretical approach, the methods used, the JOBS Program manual as well as the cooperation with the staff and the practical implementation on-site. In this context, the importance of the composition of the trainer team should be emphasized. Both professional and non-professional trainers rated the cooperation of two trainers as important and emphasized the relevance of own unemployment experiences in the trainer team. Even if it cannot be reliably depicted with the data collected, it can be assumed that the referent power, for example, is most likely to be generated by this team composition. In addition to implementation-related factors, trainers were also satisfied, on average, with the effects achieved among participants and believed that the key endpoints are being met through the training.

Some take-home messages from the trainers should be considered for future implementation: Trainers indicated that trainer workshop should be slightly longer and more face-to-face so that future trainers can adequately practice all exercises. Some trainers also mentioned that it would be helpful if the unemployed participants were better prepared for the trainings. Both issues were primarily influenced by the COVID-19 pandemic and the associated infection control measures. Due to the end of the pandemic, such difficulties should not be an issue in the upcoming recruitment and trainings. In summary, it can be concluded that the few suggestions for improvement mentioned above by the trainers can be easily put into practice.

Especially from the qualitative answers we could deduce that not all trainers were satisfied with the training manual. As the manual is crucial for the implementation of the training, a revision seems to be recommended. In addition, some trainers recommended that this rather socio-economically deprived group be provided with sufficient working utensils and catering during the training days. This could increase work ability and provide sense of appreciation and well-being.

In addition to some seemingly relatively simple “homework” that can be derived from the responses of the trainers, the predominantly positive results in all dimensions suggest that many unemployed people in Germany could benefit from continued regular implementation of the JOBS Program Germany.

### Electronic supplementary material

Below is the link to the electronic supplementary material.

Supplementary Material 1

## Data Availability

The data used for this work were collected from volunteer participants who consented to the data collection and the corresponding data protection concept. This data protection concept includes that data will not be shared with others not involved in this research project and that data collected from subjects will be deleted 5 years after completion of data collection or no later than December 31, 2028. The data protection concept was reviewed and approved by the departments responsible in the individual institutions. All data are collected, transmitted, stored and deleted in strict compliance with the German Federal Data Protection Act and the European General Data Protection Regulation (DSGVO).
