# Supplementary material for: Health promotion for the unemployed: the evaluation of the JOBS Program Germany from the trainers’ perspective
Source: Arch Public Health. 2023 Nov 17;81:199. doi: 10.1186/s13690-023-01203-2 (PMC10657034; doi:10.1186/s13690-023-01203-2)
Supplement: Supplementary file 1 — Supplementary Material 1 [file 13690_2023_1203_MOESM1_ESM.docx]

**Supplement article:**

“Health promotion for the unemployed: The Evaluation of the JOBS Program Germany from the trainers' perspective”


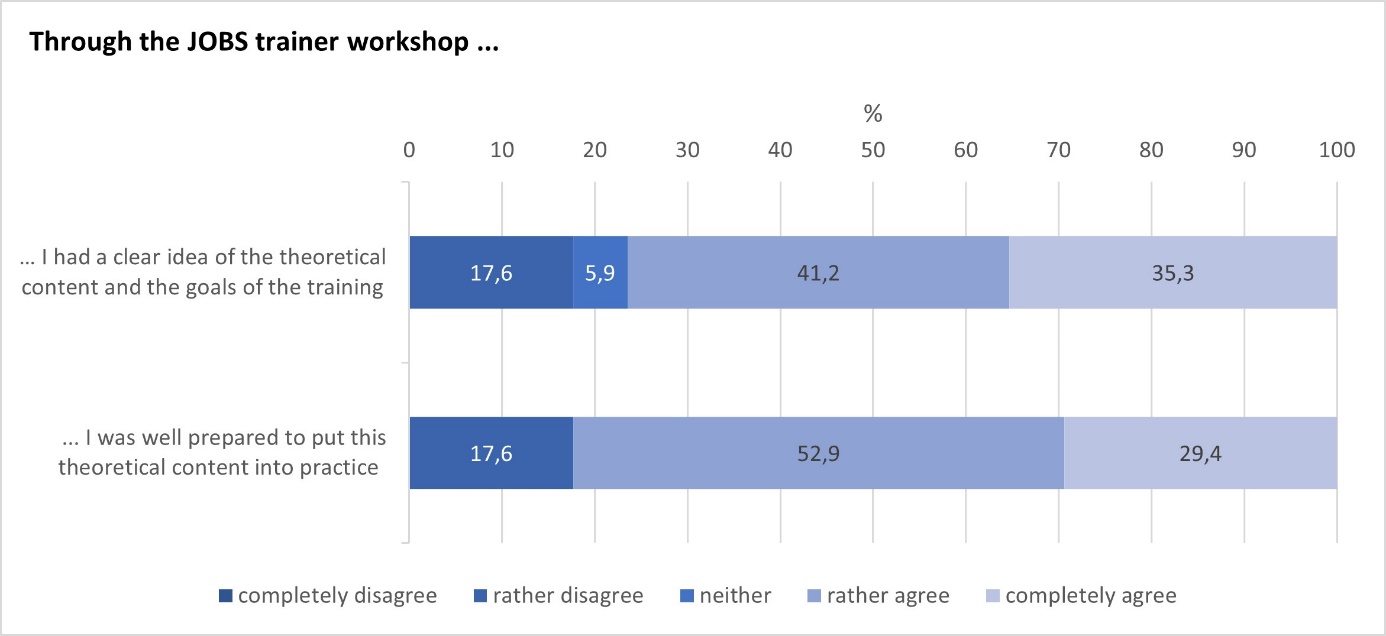


Figure 1 Trainers‘ assessment of the workshop for the JOBS Program trainers (n = 17)


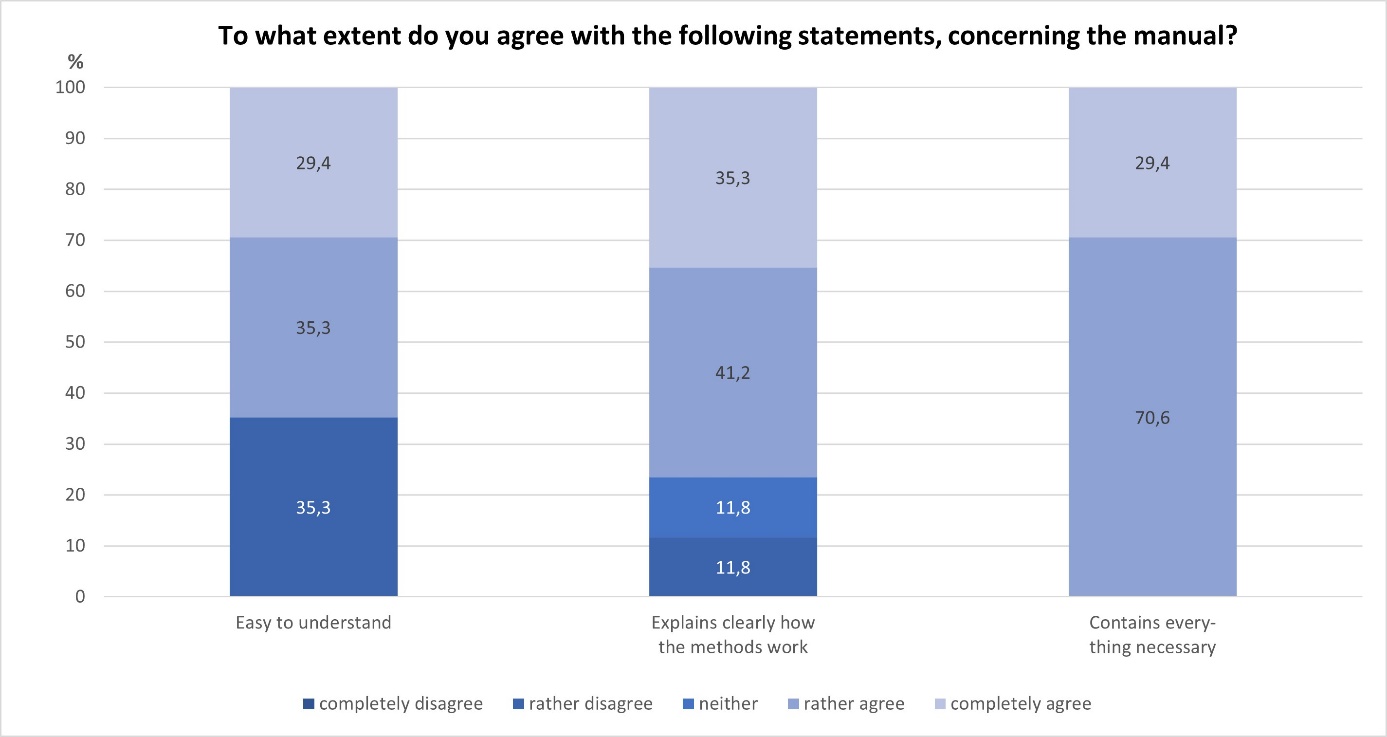


Figure 2 Trainers‘ assessment of the training manual with respect to the manual’s content (n = 17)


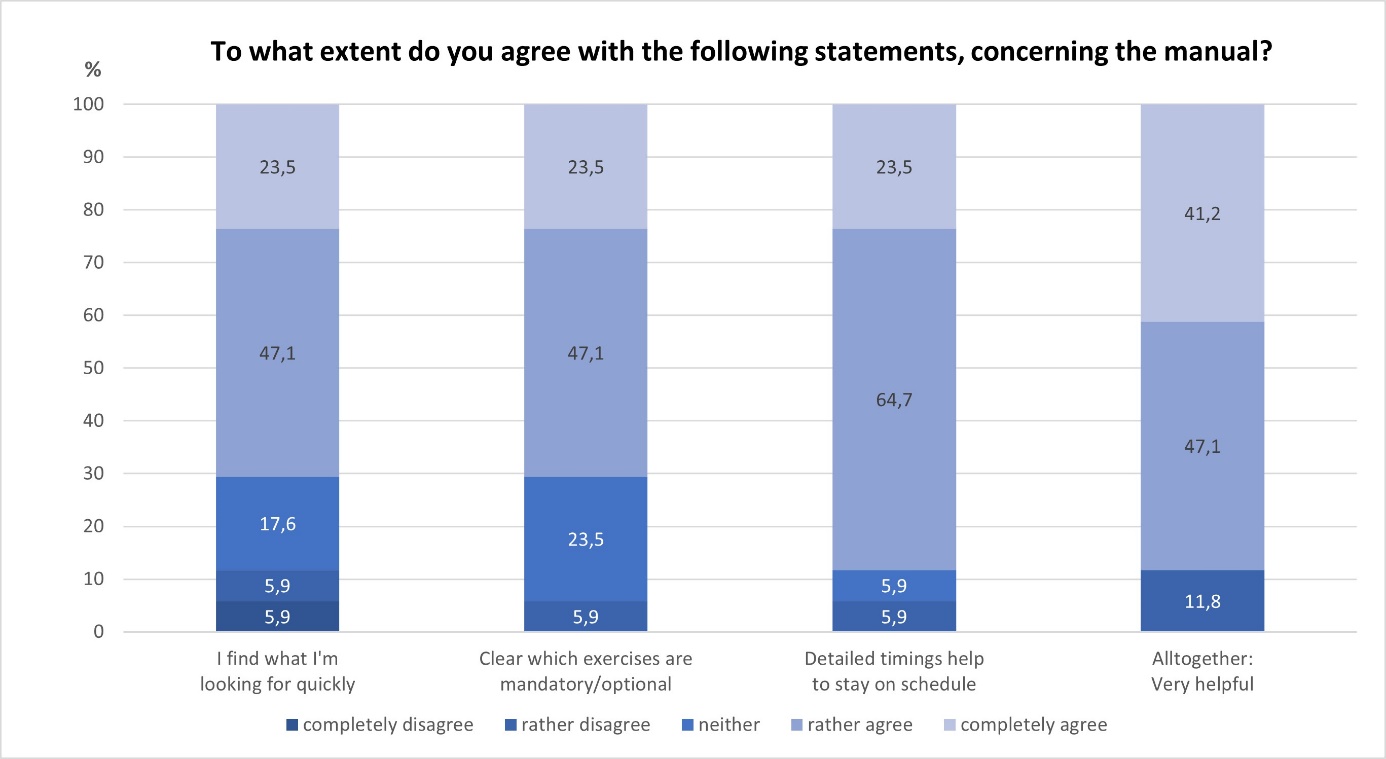


Figure 3 Trainers‘ assessment of the training manual with respect to the practical handling during the training sessions and their overall judgement of the training manual (n = 17)


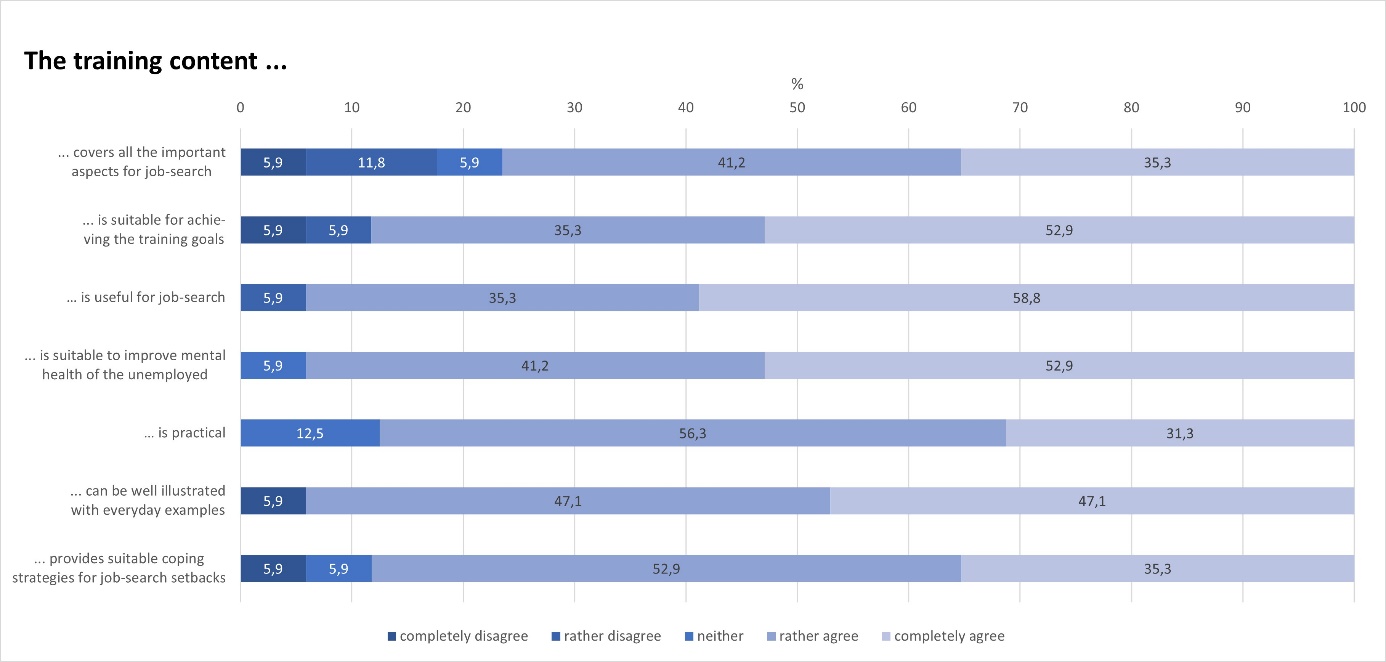


Figure 4 Trainers‘ assessment of the training content (n = 17)


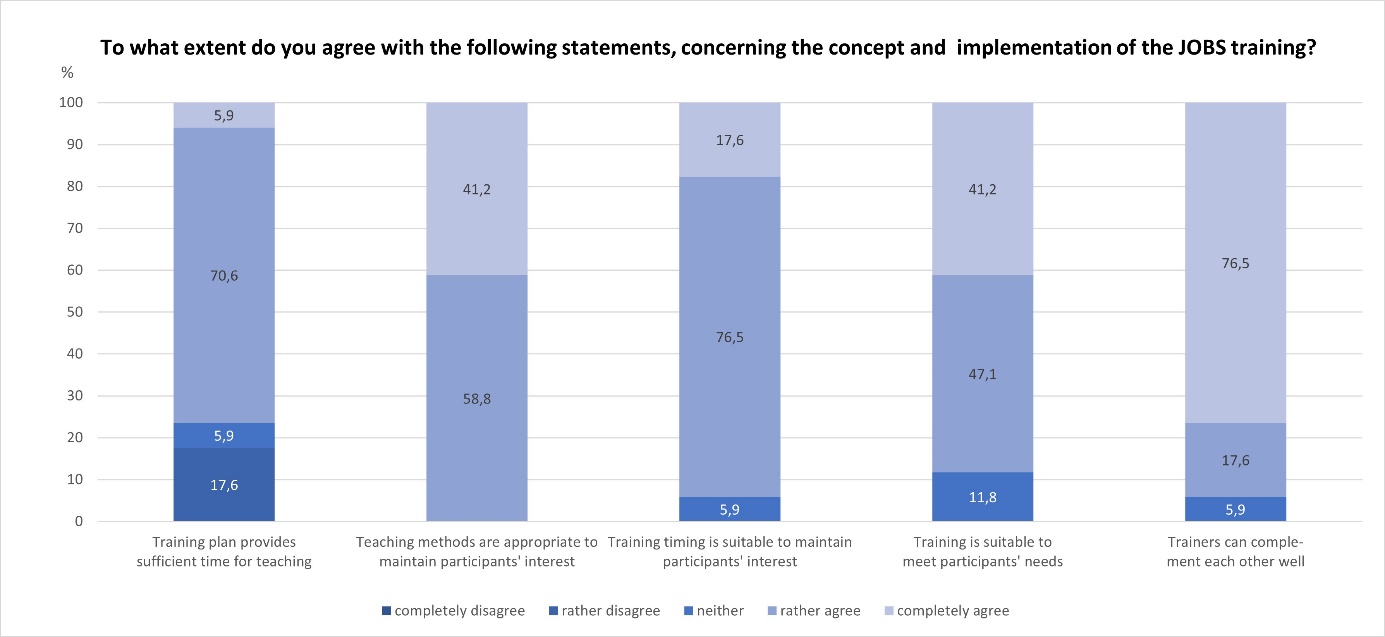


Figure 5 Trainers‘ assessment with regard to the concept and practical implementation of the JOBS training (n = 17)


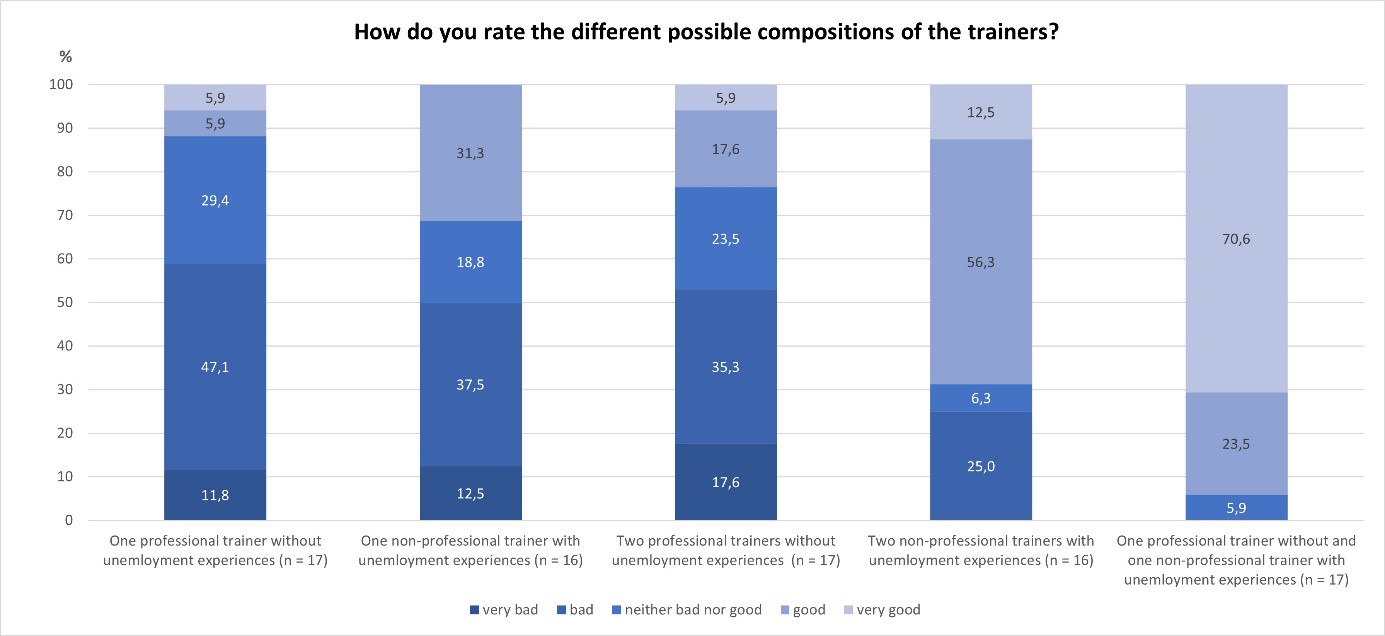


Figure 6 Trainer’s preferred choice concerning the trainer team composition (n = 17)


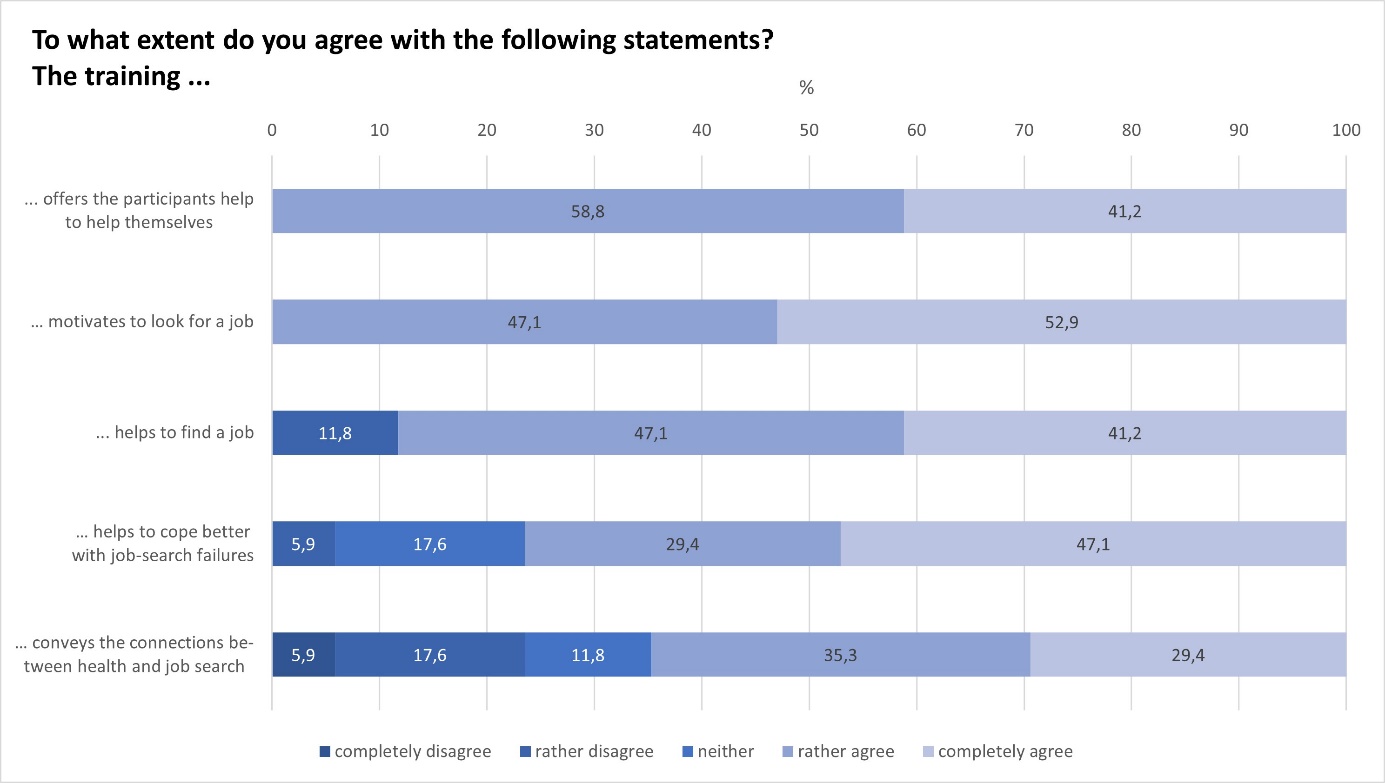


Figure 7 Trainers summarizing evaluation concerning benefits for the participants (n = 17)
